# Supplementary material for: Efficacy and safety of vapocoolant spray for vascular puncture in children and adults: A systematic review and meta-analysis
Source: PLoS One. 2023 Feb 13;18(2):e0279463. doi: 10.1371/journal.pone.0279463 (PMC9925002; doi:10.1371/journal.pone.0279463)
Supplement: S1 Table — (DOCX) [file pone.0279463.s001.docx]

**S1 Supplementary table 1. Search strategy**

Title: Efficacy and safety of Vapocoolant spray for vascular puncture in children and adults: a systematic review and meta-analysis

| **Database** | **Search strategy** | **Number of studies included** |
| --- | --- | --- |
| PubMed | ("Ethyl Chloride"[MeSH Terms] OR "Cryoanesthesia"[MeSH Terms] OR ("cold spray"[Title/Abstract] OR "Ethyl Chloride"[Title/Abstract] OR "vapocool*"[Title/Abstract] OR ("anesthe*"[All Fields] AND "skin"[Title/Abstract]) OR "1 1 1 2 tetrafluoroethane"[Title/Abstract])) AND ("injections, intravenous"[MeSH Terms] OR "catheterization, central venous"[MeSH Terms] OR "catheterization, peripheral"[MeSH Terms] OR "infusions, intravenous"[MeSH Terms] OR ("cannula*"[Title/Abstract] OR "injection*"[Title/Abstract] OR "infusion*"[Title/Abstract] OR "catheter*"[Title/Abstract] OR ("neddl*"[All Fields] AND "pain"[Title/Abstract]) OR "intravenous pain"[Title/Abstract] OR "arterial puncture"[Title/Abstract] OR "arteriopuncture"[Title/Abstract]) OR ("catheter*"[Title/Abstract] AND ("arter*"[Title/Abstract] OR "vein*"[Title/Abstract]))) AND ("random controlled trial*"[Title/Abstract] OR "placebo*"[Title/Abstract] OR "prospective"[Title/Abstract] OR "multicenter"[Title/Abstract]) | 624 |
| Web of Science | (Cold spray OR ethyl chloride OR vapocool* OR anesthe* skin OR 1,1,1,2‐tetrafluoroethane OR Cryoanesthesia OR Ethyl Chloride) AND (Infusions, Intravenous OR Injections, Intravenous OR Catheterization, Central Venous OR Catheterization, Peripheral OR cannula* OR injection* OR infusion* OR (catheter* AND (arter* OR vein* )) OR catheter* OR neddl* pain OR intravenous pain OR Arterial puncture OR arteriopuncture) AND (random* controlled trial* OR placebo* OR prospective OR multicenter OR RCT or RCTs) | 401 |
| Cochrane Library | ([Ethyl Chloride] explode all trees OR [Cryoanesthesia] explode all trees OR Cold spray OR ethyl chloride OR vapocool* OR anesthe* skin OR 1,1,1,2‐tetrafluoroethane) AND ([Infusions, Intravenous] explode all trees OR [Injections, Intravenous] explode all trees OR [Catheterization, Central Venous] explode all trees OR [Catheterization, Peripheral] explode all trees OR cannula* OR injection* OR infusion* OR catheter* OR neddl* pain OR intravenous pain OR Arterial puncture OR arteriopuncture OR catheter* AND (arter* OR vein* )) | 2960 |
| Embase | ('chloroethane'/exp OR 'cryoanesthesia'/exp OR ((cold AND spray OR ethyl) AND chloride OR vapocool* OR anesthe*) AND skin OR 1,1,1,2‐tetrafluoroethane) AND ('intravenous drug administration'/exp OR 'central venous catheterization'/exp OR 'catheterization'/exp OR (((cannula* OR injection* OR infusion* OR catheter* OR neddl*) AND pain OR intravenous) AND pain OR arterial) AND puncture OR arteriopuncture) AND (random* AND controlled AND trial* OR placebo* OR prospective OR multicenter) | 591 |
