# Supplementary material for: Efficacy and safety of vapocoolant spray for vascular puncture in children and adults: A systematic review and meta-analysis
Source: PLoS One. 2023 Feb 13;18(2):e0279463. doi: 10.1371/journal.pone.0279463 (PMC9925002; doi:10.1371/journal.pone.0279463)
Supplement: S2 Table — (DOCX) [file pone.0279463.s002.docx]

S2 Supplementary table 2. Quality assessment of studies included.

| **Study** | **Random sequence generation** | **Allocation concealment** | **Blinding of participants and personnel** | **Blinding of outcome assessment** | **Incomplete outcome data** | **Selective reporting** | **Other bias** |
| --- | --- | --- | --- | --- | --- | --- | --- |
| Mitryn[31]  et al., 2018 | Allocation: Randomized | Allocation: Randomized | Masking: Single (Participant) | Not describe. | Complete data in this study. | All outcomes specified in the methods were reported. | No evidence of other bias. |
| Armstrong[32] et al., 1990 | *The patients allocated to one of three equal-sized treatment groups using a table of random numbers.* | Not describe | Unclear | Unclear，just *All cannulations and assessments were made by the same anaesthetist* | Unclear，*One hundred and twenty female patients*  *acted*  *as the subjects in the study* | unclear | No evidence of other bias. |
| Selby [33]  et al., 1995 | *After obtaining informed consent the patients were randomly allocated to one of four groups* | Not describe. | Not describe. | Not describe. | Not describe. | Not describe. | No evidence of other bias. |
| **Study** | **Random sequence generation** | **Allocation concealment** | **Blinding of participants and personnel** | **Blinding of outcome assessment** | **Incomplete outcome data** | **Selective reporting** | **Other bias** |
| Crecelius[34]  et al., 1998 | Unclear, only wrote Subjects were randomly divided into 2 groups. | Unclear | Since most subjects would not have had previous experience with ethyl chloride, they were likely unable to determine the spray treatment they received. | The venipuncturist was not present during the spray application to reduce prejudicial behavior in catheter placement. | Unclear,*Out of the 94 patients approached, 88 patients agreed*  *to participate in the study.* | Unclear | No evidence of other bias. |
| Ramsook[35] et al., 2001 | uncler | *Based on blinded*  *randomization tables, the pharmacist provided the appropriate*  spray to the nurse caring for the patient. | Not describe. | Not describe. | *Two hundred twenty-two patients were enrolled, of whom 51%*  *were randomized to ethyl chloride and 49% to isopropyl alcohol*  *(placebo).* | Not describe. | No evidence of other bias. |
| **Study** | **Random sequence generation** | **Allocation concealment** | **Blinding of participants and personnel** | **Blinding of outcome assessment** | **Incomplete outcome data** | **Selective reporting** | **Other bias** |
| Costello[36]  et al., 2006 | *patients were assigned to one of three treatment*  *groups by random number allocation.* | *which were indistinguishable from each other except for their label as either “cannister 1”or “cannister 2.”* | *Patients were*  *blinded to the cannister’s contents.* | *The investigators and nursing staff were blinded to the cannister’s contents* | *A total of 129 subjects were approached and gave consent and assent for entry into the study. Data from two subjects were excluded from analysis due to protocol violations.* | Unclear | No evidence of other bias. |
| Robinson[37] et al., 2007 | *Random numbers were assigned to groups by the SAS (http://www.sas.com) computer program using random length blocking.* | *Sealed envelopes containing treatment*  *instructions, data sheets and study information were*  *opened only after the patient had given consent.* | Not describe. | Not describe. | *Ten patients were excluded: four for having more than two attempted cannulations, and six for incomplete data. There were no significant differences between groups in terms of withdrawals.* | Not describe. | No evidence of other bias. |
| Farion[38]  et,al.,2008 | *We randomly assigned patients to the active treatment or placebo group in blocks of 10 using a random number generator.* | *Research personnel (who were not involved in patient enrolment) masked similar canisters of active treatment or placebo, labelled them with a unique identifier and placed them in sequentially numbered opaque, sealed envelopes.* | *Next, the research assistant sprayed the cannulation site while all others in the room looked away* | *Two researchers blinded to treatment allocation independently measured the distance in millimetres between 0 and the patient’s line.* | We enrolled 80 children, randomly assigned them to the active treatment or placebo group and completed the study protocol  without deviation. | All outcomes specified in the methods were reported | No evidence of other bias. |
| France[39]  et al., 2008 | *Following consent, patients were randomized to one of*  *three groups. Block randomiza-*  *tion with a variable block size and concealment were*  *undertaken by the Research and Development Depart-*  *ment of the Royal United Hospital, Bath.* | Not describe. | Not describe. | Not describe. | *Fifty-nine patients were recruited, and AP was performed*  *by 29 different doctors.* and no one discontinued or dropped | Not describe. | Not describe. |
| **Study** | **Random sequence generation** | **Allocation concealment** | **Blinding of participants and personnel** | **Blinding of outcome assessment** | **Incomplete outcome data** | **Selective reporting** | **Other bias** |
| Hartstein[40] et al., 2008 | *Utilising a random number generator to assign subjects to the control or study group.* | *96 sequential packets containing study*  *materials, instructions for staff placing the IV cannulation and questionnaires* were prepackaged. | *We conducted an unblinded, randomised, controlled study.* | *We conducted an unblinded, randomised, controlled study.* | *Data from four of the approved subjects was not included due to incomplete documentation in one case and loss of documentation in the other three cases.* | Not describe | Skin coolant was supplied free of cost by the Gebauer  Company, Cleveland, Ohio, for use in this study. |
| Hijazi[41]  et al., 2009 | *Patients were*  *block randomised (blocks of six) by an independent*  *pharmacist using a computerised random number*  *generator.* | *These contained all the documents for data collection and a sealed envelope* | *The patients*  *were all*  *blinded to the randomisation status.* | *Staff who collected outcome data were all*  *blinded to the randomisation status.* | Unclear,*There were five protocol violations. For one patient in the control group and two in the intervention group. There were 45 patients loss to follow-up at 5 days, For 25 patient in the control group and 20 in the intervention group. but they all analysed.* | All outcomes specified in the methods were reported. | No evidence of other bias. |
| **Study** | **Random sequence generation** | **Allocation concealment** | **Blinding of participants and personnel** | **Blinding of outcome assessment** | **Incomplete outcome data** | **Selective reporting** | **Other bias** |
| Çelik[42]  et al.,2011 | *In the three consecutive dialysis sessions, every patient randomly received one of the following intervention only once before venepuncture* | Not describe. | The patients were informed about the study drugs and on how to use the visual analogue scale (VAS) for pain assessment. | *Not describe.* | The study included 41 patients undergoing conventional hemodialysis three times a week and they finished the study. | Not describe. | No evidence of other bias. |
| Waterhouse  [43] et al., 2013 | *Patients were randomized to receive either vapocoolant spray*  *or topical ice pack before IV catheter insertion*. | Not describe. | *Subjects were videotaped, and 2 independent physician observers*  *(D.R.L. and V .R.W .) reviewed all video recordings.* | Not describe. | Not describe. | Not describe. | No evidence of other bias. |
| Fossum[44]  et al., 2016 | *We used a simple randomization sequence for subject allocation.* | *Subjects understood that they would undergo random allocation to initial receipt of the intervation.* | *subjects all remained*  *blinded to subject allocation until study completion.* | *Research assistants blinded to the study interventions.* | *All subjects were eligible and included in the study. All subjects were active*  *duty military personnel.* | All outcomes specified in ClinicalTrials.gov were reported. | No evidence of other bias. |
| **Study** | **Random sequence generation** | **Allocation concealment** | **Blinding of participants and personnel** | **Blinding of outcome assessment** | **Incomplete outcome data** | **Selective reporting** | **Other bias** |
| Mace[45]  et al., 2016 | *Randomization was accomplished using a computer random num-*  *ber generator with block randomization using randomly varied block*  *sizes of 20 or 30.* | *The spray cans were supplied from outside the ED in*  *varied block sizes* | *The spray cans were not identified,*  *thus were blinded to the*  *subjects* | *The spray cans were not identified,*  *thus were blinded to*  *the research assistants applying the spray, and the health*  *care providers performing the venipuncture.* | 100 were randomized and received their allocated treatment: 50 patients received sterile water spray (placebo arm), and 50 patients received vapocoolant spray (treatment arm), and no one discontinued or dropped | All outcomes specified in the methods were reported. | No evidence of other bias. |
| Dalvandi[46] et al.,2017 | *The convenience sampling method was used and participants were randomly assigned to one of the two groups, with a blocking design*. | Not describe. | *The second main limitation of this study was lack of blindness.* | *The second main limitation of this study was lack of blindness.* | Not describe. | All outcomes specified in the methods were reported. | No evidence of other bias. |
| Edwards[47]  et al., 2017 | Unclear | *The emergency nursing staff obtained a*  *blinded, sequentially ordered study packet for*  *each enrolled patient.* | *Both the spray containers were similar in package*  *color and labeling to support study blinding.* | Both the patient and the emergency  nurse were blinded. | There was no one discontinued or dropped | All outcomes specified in the methods were reported. | No evidence of other bias. |
| Farahmand  [48] et al., 2017 | *Treatment assignment was done by block envelope randomization.* | *The researcher chose the patient's group randomly according to block envelope* | *The resident, who was not present during*  *Spray application and was unaware of spray type.* | *Neither the residents nor the patients knew which kind of intervention had been*  *performed.* | All the patients who entered completed the study. | All outcomes specified in the methods were reported. | No evidence of other bias. |
| Mace[49]  et al., 2017 | *Randomization was accomplished using a computer random num-*  *ber generator with block randomization using randomly varied block*  *sizes of 20 or 30.* | *The spray cans were supplied from outside the ED in*  *varied block sizes* | *The spray cans were not identified,*  *thus were blinded to the*  *subjects* | *The spray cans were not identified,*  *thus were blinded to*  *the research assistants applying the spray, and the health*  *care providers performing the venipuncture.* | Of the 544 patients recruited to the study, 300 consented, were  randomized and received their allocated treatment: 150 patients  received sterile water spray (placebo arm) and 150 patients  received vapocoolant spray (treatment arm),and no one discontinued or dropped | All outcomes specified in the methods were reported. | No evidence of other bias. |
| **Study** | **Random sequence generation** | **Allocation concealment** | **Blinding of participants and personnel** | **Blinding of outcome assessment** | **Incomplete outcome data** | **Selective reporting** | **Other bias** |
| Rusch [50]  et al., 2017 | *Randomization on the basis of*  *random numbers from www.random.org.* | *After arriving in the operating room*  *area, patients having elective surgery were allocated*  *according to instructions from a sealed, non-*  *transparent envelope.* | *During the procedure, doctor and patient communicated in a structured and standardized way, with the patient blinded as well as possible.* | Not describe. | *The number of and reasons for the 8 cases of*  *non-adherence to the study protocol (flow chart).*  *Because of this small number the results of the per-*  *protocol analysis (n=442) hardly differed from the*  *intention-to-treat analysis (n=450).* | All outcomes specified in the methods were reported. | No evidence of other bias. |
| Barbour [51]  et al., 2017 | *Block randomization was done using randomly varied block sizes of 20 or 30 by a computer random number generator.* | Unclear,*The application of spray was performed via the recommended technique by a trained research assistant.* | *Participants*  *were blinded to the contents.* | *Research assistants, and health care providers were blinded to the contents.* | Complete data in this study. | All outcomes specified in the questionnaires were reported. | No evidence of other bias. |
| **Study** | **Random sequence generation** | **Allocation concealment** | **Blinding of participants and personnel** | **Blinding of outcome assessment** | **Incomplete outcome data** | **Selective reporting** | **Other bias** |
| DHami[52]  et al., 2020 | *Randomisation was done using computer generated random list.* | *Allocation concealment was performed by sequentially arranged opaque sealed envelopes.* | *Participants and the primary investigator were not blinded.* | *Another investigator which was blinded to the procedure measured*  *the pain experienced* | Discontinued/dropped  N=0 | All outcomes specified in the methods were reported. | No evidence of other bias. |
| Basak[53]  et al., 2021 | *The e-PICOS biostatistics software program (MedicReS,*  *New York, NY) was used for randomization.* | Unclear,*The participants*  *were assigned to the groups by the primary researcher in*  *the order of their arrival.* | Unclear | *The second researcher, who did*  *not know which group each participant belonged to, made*  *a face-to-face assessment.* | This study randomized 88 donator, and they all finished the study | The primary outcome same as ClinicalTrials.gov | No evidence of other bias. |
| Supan[54]  et al., 2021 | *All participants were randomized equally (n=62)*  *into three groups using a sealed envelope technique* | *All participants were randomized equally (n=62)*  *into three groups using a sealed envelope technique* | Not describe. | Not describe. | *Four patients in group A, four in group B, and two in group C were dropped out due to the procedural failure at the first attempt.* | Not describe. | No evidence of other bias. |
| Ghasemi[55]  et al.,2022 | *Six blocks were defined (ABC,ACB, BAC, BCA, CAB, and CBA) and a number between 1 and 6 were assigned to each block. By rolling dice, the*  *sequence of blocks was determined.* | *The first author and the statistician were not present in the patient enrollment*  *and assignment (allocation was concealed)* | *Due to the nature of the intervention blinding was not possible.* | *all subjects and pain measurement was conducted by two*  *members of the research team who were not present at*  *blocking concealment, and analysis.* | *In both arms one child was withdrawn due*  *to the disinterest of parents, then both arms had 17 subjects in the second measurement. Arm (A) lost 2 children and arm (B) lost 1 child in the third session. In the end,31 children completed all three measurements and their data was analyzed with ANOVA.* | All outcomes specified in the methods were reported | No evidence of other bias. |

^RCT: randomized controlled trial^
