# Supplementary material for: Efficacy and safety of vapocoolant spray for vascular puncture in children and adults: A systematic review and meta-analysis
Source: PLoS One. 2023 Feb 13;18(2):e0279463. doi: 10.1371/journal.pone.0279463 (PMC9925002; doi:10.1371/journal.pone.0279463)
Supplement: S3 Table — (DOCX) [file pone.0279463.s003.docx]

Supplementary Table 3 Summary results of regression analysis

| Outcomes | Coef | Standard Error | t | P>\|t\| | 95% CI | |
| --- | --- | --- | --- | --- | --- | --- |
| Age | 0.1039 | 0. 1853 | 0.56 | 0.586 | -0.3039 | 0.5116 |
| Distance | 0.4593 | 0.1370 | 3.35 | 0.006 | 0.1577 | 0.7608 |
| Time | -0.3461 | 0.1336 | -2.59 | 0.025 | -0.6402 | -0.0519 |
| Vapocoolant type | 0.1310 | 0.1235 | 1.06 | 0.312 | -0.1410 | 0.4030 |
