# Supplementary material for: Efficacy and safety of vapocoolant spray for vascular puncture in children and adults: A systematic review and meta-analysis
Source: PLoS One. 2023 Feb 13;18(2):e0279463. doi: 10.1371/journal.pone.0279463 (PMC9925002; doi:10.1371/journal.pone.0279463)
Supplement: S4 Table — (DOCX) [file pone.0279463.s004.docx]

S4 Supplementary table 4. Assessment of publication bias.

| Outcome | No. of studies | t | p |
| --- | --- | --- | --- |
| Pain scores after venipuncture | 16 | 1.77 | 0.099 |
| First attempt success rate | 14 | 0.03 | 0.979 |
